# Remotely controlled mandibular positioning of oral appliance therapy during polysomnography and drug-induced sleep endoscopy compared with conventional subjective titration in patients with obstructive sleep apnea: protocol for a randomized crossover trial

**DOI:** 10.1186/s13063-019-3698-4

**Published:** 2019-10-29

**Authors:** Marijke Dieltjens, Marc J. Braem, Sara Op de Beeck, Anneclaire V. M. T. Vroegop, Elahe Kazemeini, Eli Van de Perck, Jolien Beyers, Chloé Kastoer, Kristien Wouters, Marc Willemen, Johan A. Verbraecken, Olivier M. Vanderveken

**Affiliations:** 1 Translational Neurosciences, Faculty of Medicine and Health Sciences, University of Antwerp, Wilrijk, Antwerp Belgium; 2 Department of Otorhinolaryngology, Head and Neck Surgery, Antwerp University Hospital, Wilrijkstraat 10, 2650 Edegem, Antwerp Belgium; 3 Department of Special Dentistry Care, Antwerp University Hospital, Edegem, Antwerp Belgium; 4 Multidisciplinary Sleep Disorders Centre, Antwerp University Hospital, Edegem, Antwerp Belgium; 5 Clinical Trial Center, Antwerp University Hospital, Edegem, Antwerp Belgium; 6 Department of Pulmonology, Antwerp University Hospital, Edegem, Antwerp Belgium; 7 Laboratory of Experimental Medicine and Pediatrics (LEMP), Faculty of Medicine and Health Sciences, University of Antwerp, Wilrijk, Antwerp Belgium

**Keywords:** Mandibular advancement device, Sleep-disordered breathing, Apnea–hypopnea index

## Abstract

**Background:**

The amount of mandibular protrusion is a key factor in optimizing the efficacy of mandibular advancement device (MAD) therapy in an individual patient diagnosed with obstructive sleep apnea. This process is called titration and is generally based on resolution of subjective symptoms like snoring and/or daytime sleepiness as a function of protrusion. An objective approach uses a remotely controlled mandibular positioner (RCMP) during a full-night polysomnography (PSG), in analogy with continuous positive airway pressure (CPAP) titration. More recently, the feasibility of RCMP use during drug-induced sleep endoscopy (DISE) titration was reported.

**Methods:**

This randomized crossover trial will compare DISE-assisted titration to PSG-guided titration, as well as with the conventional subjective titration method. The primary outcome is the actual mandibular protrusive position found to be the most optimal for each tested titration procedure. Furthermore, the therapeutic efficacy will be compared among the different titration modalities using level 1 sleep studies.

**Discussion:**

Currently, the optimal titration of MAD therapy is most often based on ‘trial and error’. The conventional method relies on subjective improvement in symptoms, although this may not provide the most accurate indicator for efficient titration. Therefore, relying on objective criteria in the titration process should be advantageous. In analogy with CPAP, titration of the most optimal mandibular protrusion could be performed using RCMP during an overnight titration PSG. Recently, it was shown that titration under direct visualization of upper airway patency and collapsibility is feasible using the RCMP during DISE. However, no clinical results for such a procedure are as yet available. This study is the first to compare the most optimal mandibular protrusive position according to three titration procedures, as well as to compare the therapeutic efficacy of these titration methods.

**Trial registration:**

ClinicalTrials.gov, NCT03716648. Registered on 23 October 2018.

## Background

Obstructive sleep apnea (OSA) is a prevalent public health issue, affecting up to 17% of adult women and 34% of adult men [1]. It is characterized by repetitive episodes of partial (hypopneas) or complete (apneas) upper airway obstruction during sleep, leading to nocturnal hypoxemia and sleep fragmentation [2]. The clinical daytime consequences associated with untreated OSA include excessive daytime sleepiness (EDS), impaired cognitive performance and reduced quality of life. There is a strong correlation between OSA and both traffic and occupational accidents [3]. Furthermore, epidemiological studies provide objective evidence that OSA is an independent risk factor for cardiovascular morbidity and mortality [4–6].

The diagnostic work-up for OSA follows both subjective and objective appraisal [7]. It starts with obtaining a medical history with an emphasis on subjective symptoms, followed by a clinical assessment and an overnight sleep study for the objective diagnosis. The severity of OSA is traditionally expressed by the apnea–hypopnea index (AHI), defined as the number of apneas and hypopneas per hour of sleep [8]. Based on the AHI, the severity of OSA can be described as mild (5 ≤ AHI < 15/h), moderate (15 ≤ AHI < 30/h) and severe OSA (AHI ≥ 30/h) [8].

Due to the socioeconomic consequences and the related morbidities and mortality, adequate treatment of OSA is important. Currently, several treatment modalities are available, but in general the treatment is a stepwise approach starting with behavior modifications indicated for all patients with a modifiable risk factor. The most important conservative measure in the management of OSA is weight loss and should be advised to all overweight or obese OSA patients, as significant decrease in body weight is associated with reduced OSA severity: a reduction in weight of 10% is associated with an improvement in AHI of 26% [9]. However, it is recommended to combine weight loss and other conservative measures with nonsurgical or surgical treatment options [7].

Therapy with continuous positive airway pressure (CPAP) is the standard nonsurgical treatment option for patients with moderate to severe OSA [10]. CPAP therapy prevents upper airway collapse by providing a pneumatic splint using a constant positive pressure at the level of the collapsible segment of the upper airway throughout the respiratory cycle [11]. The amount of pressure required to avoid upper airway collapse cannot be predetermined based on OSA severity and needs to be assessed individually. To achieve the optimal pressure alleviating the disease, the current standard practice involves an overnight attended CPAP titration polysomnography (PSG) in which the applied pressure is manually increased by a sleep technician each time respiratory events occur [12]. Rather than the use of an overnight attended PSG to find the optimal CPAP pressure, an unattended auto-titrating CPAP device can be used for several nights [13, 14]. Auto-titrating CPAP devices continuously monitor the patient’s snoring, airflow and flow limitation, and provide variable CPAP pressures in accordance with respiratory events using an algorithmic approach. As such, these devices can be used either to treat a patient with different pressures delivered during different sleeping conditions, in response to differing degrees of upper airway obstruction in these states, or to identify the most appropriate fixed CPAP pressure level. More recently, Civelek et al. [12] proposed to titrate the CPAP pressure under direct visualization of upper airway collapse during drug-induced sleep endoscopy (DISE). DISE enables a dynamic, three-dimensional, real-time evaluation of the anatomical sites of upper airway collapse during drug-induced sleep which ideally mimics natural sleep, although without REM sleep being present [15]. This DISE-assisted titration showed comparable results regarding the optimal CPAP pressure that alleviates OSA as compared to the conventional titration PSG. Overall, once the optimal pressure is determined, CPAP therapy is highly efficacious in terms of alleviation of OSA severity. However, the high clinical efficacy can be compromised by low patient acceptance or suboptimal adherence, thereby limiting the overall clinical effectiveness of this therapy [16].

Another nonsurgical OSA therapy is the use of an oral appliance that is worn intraorally at night, finding retention on the teeth, and aimed at reducing upper airway collapse by protruding the mandible. Such an appliance will further be referred to as a ‘mandibular advancement device’ (MAD). Several studies in the literature compared the efficacy of MAD therapy with CPAP, showing that both treatment modalities improve the OSA severity. Although CPAP shows a greater efficacy compared to MAD therapy, comparative effectiveness and health outcomes between CPAP and MAD therapy are found. This might be explained by greater efficacy of CPAP being offset by inferior compliance relative to MAD [17].

Many designs exist, but a custom-made MAD that includes a titratable mechanism allowing for gradual protrusion is currently the preferential type [18, 19].

Based on the literature, the amount of protrusion seems to be a key factor in optimizing MAD efficacy, although more protrusion does not always yield better results [20]. Therefore, the optimal mandibular protrusion for MAD therapy needs to be determined in the individual patient and adjusted in terms of tolerability versus efficacy [21, 22]. However, up to now, no proven standard exists on how to determine this optimal MAD protrusion. So, at this stage, MAD titration remains rather ‘trial and error’ [23].

Most outcome studies on MAD therapy are using a so-called ‘subjective titration protocol’, relying on both the physical limits of the patient’s mandibular protrusion and the self-reported evolution of symptoms, such as snoring and/or daytime sleepiness [24–27]. However, such subjective improvement in symptoms may not provide the most accurate indicator for efficient titration of the MAD: it may result in a suboptimal treatment outcome, since the reduction of the subjective complaints may encourage a premature interruption of the titration [28, 29]. In an approach analogous to a CPAP titration night, the mandible could be progressively advanced during sleep, each time respiratory events occur. A so-called ‘remotely controlled mandibular positioner’ (RCMP) has been applied in overnight sleep studies to prospectively determine the optimal mandibular protrusion for MAD treatment in individual patients [21, 23, 30–32]. The literature has shown a greater reduction in AHI after RCMP titration as compared to conventional titration methods [30].

As part of the pretreatment work-up when non-CPAP treatment options are considered, DISE allows for a dynamic assessment of upper airway behavior during induced sleep, disclosing the site(s) of upper airway obstruction [33]. Recently, it was shown that the application of an RCMP during DISE is feasible and that it can be used to prospectively determine the targeted mandibular protrusion [34].

The present article proposes the protocol for a clinical study consisting of a prospective, randomized, crossover trial comparing the most optimal protrusive mandibular position as well as the treatment outcome in terms of AHI, using these three titration procedures for MAD therapy.

## Methods

### Aim, design and setting

This clinical trial proposes a prospective, randomized, crossover trial in patients diagnosed with OSA and referred for MAD treatment. Patients will be recruited from the Multidisciplinary Apnea and Snoring Clinic of the Antwerp University Hospital (Edegem, Belgium).

This study aims to compare the optimal mandibular protrusion values obtained during three titration procedures in each individual patient performed in randomized order: titration of the MAD in the home setting during 1 month based on both the physical limits of the patient’s mandibular protrusion and the resolution of subjective complaints, such as socially disturbing snoring and daytime somnolence, as currently used in routine clinical practice; an overnight titration PSG using the RCMP with stepwise mandibular protrusion until respiratory events are reduced; and titration of the mandible during DISE using the RCMP until upper airway collapse at all collapsible levels is eliminated. The DISE-assisted titration will take on average 30 min with a maximum duration of 45 min and will be carried out by an ear, nose and throat (ENT) surgeon experienced in DISE assisted by a dental sleep professional. After each type of titration, PSG-guided or DISE-assisted, respectively, in randomized order, the patient will use the MAD for 1 month in the obtained protrusion relative to the respective method. The subjective titration will also be done during a 1-month period.

**Fig. 1:**
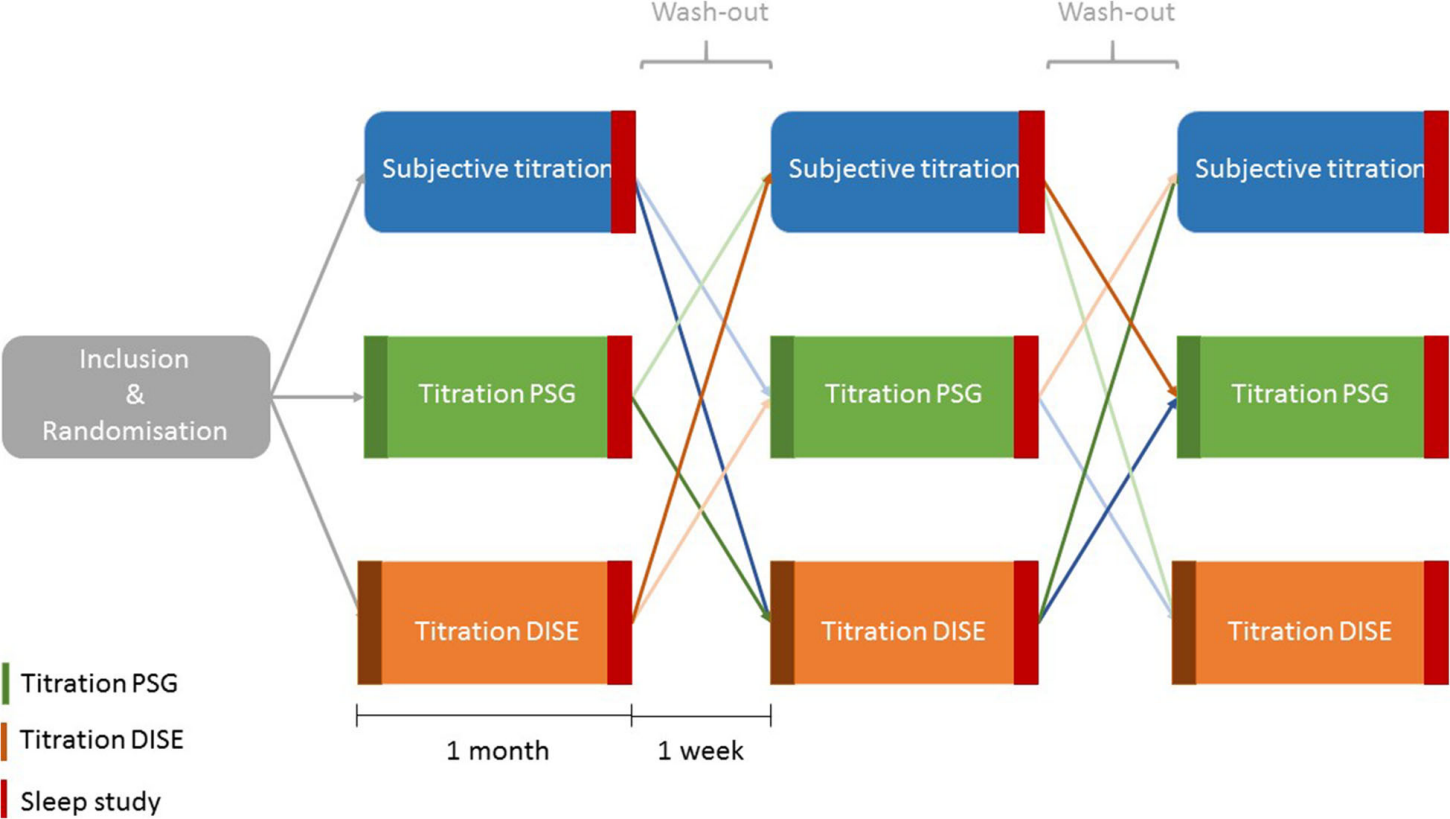
Schematic overview of the study design. Patients will undergo three titration procedures —subjective titration (green), titration PSG (orange) and, titration DISE (yellow) — in randomized order. This leads to six different possible sequences. A 1-week washout period is integrated between the different titration procedures. A follow-up sleep study is performed at the end of each titration method. DISE, drug-induced sleep endoscopy; MAD, mandibular advancement device; PSG, polysomnography

**Fig. 2:**
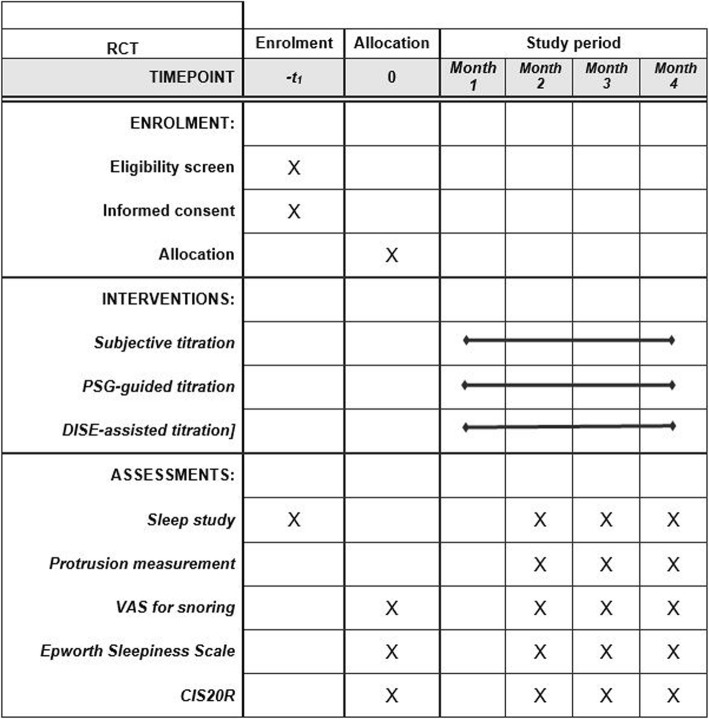
Schedule of protocol assessments following Standard Protocol Items: Recommendations for Interventional Trials (SPIRIT). Interventions and assessments will be administered at different time points (indicated by X). See text for more details. CIS20R, Checklist Individual Strength; DISE, drug-induced sleep endoscopy; PSG, polysomnography; RCT, randomized crossover trial; VAS, visual analog scale

A follow-up sleep study will be performed after each procedure for evaluation of the efficacy of the MAD in terms of reduction of AHI. A washout interval of 1 week between the different test conditions is integrated into the protocol. The flowchart of the protocol is shown in Figs. 1 and 2.

### Participants

Patients diagnosed with moderate to severe OSA and referred to the special care dentistry unit for treatment with an MAD will be recruited for participation in this study and will be asked for informed consent prior to the start of the study. To ensure accurately diagnosed OSA, all patients will need an initial baseline PSG of maximally 2 years old prior to the effective start of treatment, and show stable body weight (± 5 kg) since that PSG. No ENT surgery may be performed in eligible patients since the diagnostic PSG. An ENT examination, a dental screening and an appraisal of inclusion and exclusion criteria (see later) will be performed.

#### Inclusion criteria

- ◦ Patients with moderate to severe OSA (obstructive apnea/hypopnea index (oAHI) ≥ 15/h)
- ◦ Age > 18 years
- ◦ PSG < 2 years old with stable body weight (± 5 kg) and no ENT surgery since the diagnostic PSG; if body weight changes significantly or ENT surgery is performed, a new diagnostic PSG is required to confirm the diagnosis of moderate to severe OSA
- ◦ Normal clinical and radiological (including orthopantogram X-ray), periodontal and temporomandibular joint examination
- ◦ Subject is capable of giving informed consent
- ◦ No documented abuses (alcohol, drugs, etc.)

#### Exclusion criteria

- ◦ Edentulous patients
- ◦ Insufficient teeth to support MAD
- ◦ Active periodontal problems including tooth mobility
- ◦ Active temporomandibular joint dysfunction
- ◦ Limited maximum protrusive capacity (< 6 mm)
- ◦ Limited vertical opening (< 25 mm)
- ◦ Enlarged palatine tonsils (Friedman grade IV tonsils)
- ◦ Degenerative neuromuscular disorders
- ◦ Pregnancy

### Study protocol

After inclusion, each patient will undergo three different titration procedures of the protrusive mandibular position, in a randomized order: subjective titration; RCMP titration during DISE; RCMP titration during PSG (see Fig. 1).

**Fig. 3:**
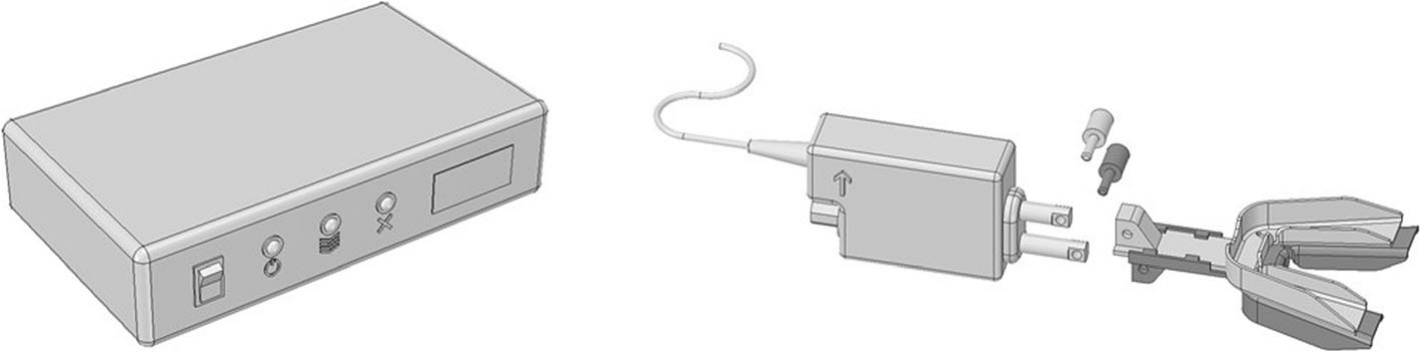
Schematic overview of the commercially available remotely controlled mandibular positioner (RCMP) device (MATRx™; Zephyr Sleep Technologies Inc., Calgary, Canada). Left: the controller that receives commands from the device software. Right: the motorized RCMP attached to disposable upper and lower dental impression trays. This schematic overview was drawn by our research group

In our study, a commercially available RCMP (MATRx™; Zephyr Sleep Technologies Inc., Calgary, Canada) will be used [21]. The RCMP uses dental trays filled with high-viscosity impression material that fit retentively over the teeth to allow for progressive titration of the mandible during sleep, without arousing the patient. It consists of a controller that receives commands from the device software and, in turn, activates a stepping motor attached to dental trays in the patient’s mouth (Fig. 3). The positioner (50 g; 62 mm × 41 mm × 20 mm) has two movable rods that connect to brackets extending anteriorly from the dental trays. The upper rod is driven by an internal linear actuator and attaches to the upper bracket. The lower rod is driven by a manually adjustable screw and connects to the lower bracket [21]. Before the PSG-guided or DISE-assisted titration is planned, the dental sleep professional will fit upper and lower disposable dental impression trays that attach to the RCMP positioner. During that visit, the dentist will record the habitual bite position, maximum retrusion and maximum protrusion as voluntarily performed during wakefulness. These measurements will be used to set the individual’s mandibular range of motion for the RCMP studies.

#### Subjective titration

After fitting the MAD, there is a 1-month period during which patients titrate their MAD. Each patient will individually be instructed and trained to perform the actual titration of the MAD. In general, the degree of mandibular advancement is progressively increased until a significant improvement or resolution of symptoms occurs, or until the patient cannot tolerate any further advancement.

#### Titration during drug-induced sleep endoscopy (DISE) — DISE-assisted titration

The DISE will be performed by an ENT surgeon experienced in DISE in order to visualize the dose-dependent effect of the mandibular protrusion on the upper airway collapsibility. The DISE is performed in a semi-dark and silent operating theater with the patient lying in a supine position. The different collapsible levels of the upper airway that can be investigated during DISE are the palate (velopharynx), the oropharynx, the tongue base, the hypopharynx and the epiglottis. The degree of collapse at each level is reported as none, partial or complete. The pattern of the pharyngeal collapse during the obstructive events is classified as concentric, anteroposterior and/or laterolateral [35].

Upon connection of the RCMP to a dedicated laptop, calibration of the actual versus the software-guided protrusion is verified using the RCMP ruler and proprietary developed software to rule out day-to-day variation caused by environmental factors such as room temperature or humidity. This procedure ensures the mandibular displacement is read out correctly from the ruler present on the upper tray fitted over the tooth arcs (Fig. 3). After the calibration, the RCMP trays are fitted in an edge-to-edge position to avoid excessive muscle tension. A flexible fiberoptic nasendoscope (Type ENF-GP; Olympus Europe GmbH, Hamburg, Germany) will be introduced by the ENT surgeon in the awake patient to evaluate the awake upper airway state. Thereafter, sedation will be induced by intravenous administration of midazolam (bolus injection of 1.0–2.0 mg) and propofol using a target-controlled infusion system (2.0–3.0 μg/ml mean effect site concentration). The transition to unconsciousness similar to stage 2 sleep is aimed at and examined by ensuring an absence of patients’ eyelash reflex after stimulation by means of a gentle brush. Findings are noted using a uniform upper airway scoring system evaluating the level of snoring, presence of apneas, degree of oxygen saturation, degree and configuration of obstruction(s) and the level of upper airway collapse [36].

When target sedation is reached, examined by ensuring an absence of patients’ eyelash reflex after stimulation by means of a gentle brush, the RCMP will be remotely protruded in increments of 2 mm in response to the visualized upper airway collapse at the different collapsible levels until a stable upper airway together with an absence of oxygen desaturation and snoring is reached.

If a stable upper airway without oxygen desaturation and snoring is noted, the mandible will be remotely retruded for 1 mm. If the upper airway remains stable, further so-called ‘reversed titration’ will continue. This approach will be repeated until the effective target protrusive position can be determined, defined as the minimal mandibular threshold position corresponding to a stable upper airway in the absence of snoring, oxygen desaturation and apneas. When a stable upper airway is reached, the titration procedure will be fine-tuned in smaller steps (0.5 mm) to be as precise as possible.

After every protrusive or retrusive movement, the RCMP protrusion will be checked on the RCMP ruler versus the protrusion measured by the software, ensuring correct positioning of the mandible during the upper airway assessment.

If the DISE is scored as ‘predicted success’, the predicted effective target protrusive position is provided to the dentist to start MAD therapy in that position. If the DISE-assisted titration is inconclusive or scored as ‘predicted failure’, the MAD will be set at the most optimal protrusive position with the most open and stable upper airway following the medical doctor performing the DISE procedure. If the DISE procedure is inconclusive in the entire range of motion, the MAD will be set at 75% of maximal protrusion, in analogy with PSG-guided titration.

#### Titration during polysomnography (PSG) — PSG-guided titration

The mandibular protrusion titration PSG will be performed using the RCMP device, MATRx, during a standard type I, full-night, attended PSG. The PSG recordings and the scoring are performed using the AASM 2012 rules [8].

At the start of the PSG, the participant’s dental trays are attached to the mandibular positioner of the RCMP device, and the positioner is calibrated to the PSG system using the device software as explained earlier.

The titration procedure itself was previously described by Remmers et al. [21]. Once in stage 2 sleep based on the PSG signals, the patient’s mandible will be protruded remotely in 0.5-mm steps in response to evidence of apneas and/or hypopneas. If an electroencephalographic (EEG) arousal occurs, no further advancement will be attempted until stable sleep resumes. Stepwise mandibular protrusion will continue until respiratory events are reduced to normal in all sleep stages, in both supine and lateral positions, or until maximal protrusion is reached. Afterwards, an independent researcher will score the PSG together with body position and mandibular position signals, and will predict success or failure with MAD therapy, based on the predetermined interpretative rules described by Remmers et al. [21].

The patient should have REM sleep for ≥ 5 min in the supine position or in the lateral position if REM in the supine position was not observed. All REM cycles will be evaluated to identify a minimum 5-min interval where ≤ 1 respiratory event occurs. If this is the case, the PSG will be scored as ‘predicted MAD success’ for that protrusive position. If not, the PSG will be judged to predict ‘therapeutic failure’. The predicted effective target protrusive position is the minimum protrusive position that is associated with ≤ 1 respiratory event per 5-min REM sleep.

If the PSG is scored as ‘predicted success’, the predicted effective target protrusive position is provided to the dentist to start MAD therapy in that position. If the PSG is scored as ‘predicted failure’, an alternative position equal to 75% of maximal protrusion is provided. If the PSG-guided titration was scored as ‘inconclusive’ due to a lack of REM sleep, this will be incorporated into our database, but the MAD will be set at the most effective protrusive position found in non-REM sleep.

#### Sleep study

A level 1 sleep study will be planned at baseline and to assess the efficacy of the MAD treatment in the titrated positions. For the DISE-assisted and PSG-guided titration, the patients will use the MAD in the obtained protrusion during 1 month before the follow-up sleep study, while the subjective titration will be performed during a 1-month period directly followed by the follow-up sleep study (see also Fig. 1). A washout period of at least 1 week is inserted between the polysomnographic evaluation of the protrusive position obtained during a titration procedure and the start of the next titration procedure.

The follow-up sleep study will be scored by an independent sleep technician who is blinded to the treatment phase.

The results of the different polysomnographic evaluations will be discussed with the patient by the multidisciplinary team, together with an interview about the patient’s experience of snoring and daytime sleepiness and possible side effects of MAD treatment.

#### Measurement of MAD adherence

A temperature-sensitive microsensor with on-chip integrated read-out electronics (Theramon®; Handels- und Entwicklungsgesellschaft, Handelsagentur Gschladt, Hargelsberg, Austria) will be embedded in the MAD on the upper right side. Objective compliance measurement is based on the assumption that the OA is worn when a temperature ≥ 35 °C is recorded. At every follow-up visit, the objective MAD adherence will be collected.

### Outcome measures

#### Primary outcome

The protrusive position that was predicted as the effective target protrusive position during each titration method (subjective titration, PSG-guided titration PSG and DISE-assisted titration) will be assessed and compared between the different titration methods.

#### Secondary outcomes

Efficacy of the MAD therapy will be assessed as a measure of treatment outcome by comparing the baseline AHI with the AHI under MAD therapy.

In addition, structured questionnaires to evaluate tolerance and side effects, subjective snoring, daytime sleepiness and fatigue will be completed by all patients at baseline and at follow-up evaluations.

The degree of daytime sleepiness will be assessed using the Epworth Sleepiness Scale (ESS) [37]. A standard 10-point visual analog scale (VAS) for the scoring of snoring as assessed by the bed partner will be used only in patients who have a bed partner who is able to report the snoring intensity. This VAS is ranging from 0 to 10, with 0 equaling no snoring and 10 causing the bed partner to leave the room or sleep separately [38]. Heavy snoring will be defined as a VAS snoring index of at least 7. Fatigue severity will be measured by the Checklist Individual Strength (CIS20R) [36]. The CIS20R, and more specifically its subscale on fatigue, is a standardized and validated questionnaire that consists of eight items scored on a 7-point Likert scale. The scores range from 8 (normal fatigue) to 56 (most severe fatigue). A score of 26 or lower indicates a normal energy level, scores between 27 and 35 indicate mild fatigue and a score of 36 or higher indicates severe fatigue.

For all secondary outcomes, non-inferiority of RCMP titration with PSG compared to RCMP titration with DISE will be assessed. Additionally, differences between subjective titration and both RCMP titration protocols will be investigated.

### Statistics

#### Sample size calculation

The primary endpoint of this study protocol is to assess equivalence of the protrusive position obtained with the three titration protocols. We need 65 patients to have 80% power to show equivalence of two procedures with an equivalence margin of 1.6 and a standard deviation of 3.7. These numbers are based on the results of Civelek et al. [12]. The significance level of 0.05 will be Bonferroni corrected to 0.0167, to account for comparing three titration protocols.

For every patient dropping out of the study before the first visit, an extra patient will be recruited. Based on previous studies, the dropout rate is anticipated to be around 20%, so we expect to include 78 patients in total.

#### Statistical analyses

The primary endpoint and all continuous secondary endpoints will be analyzed using a linear mixed-effects model with period, sequence and procedure as fixed effects, and a patient nested within sequence as a random effect. For the response rate, a logistic mixed-effects model with the same specifications will be used. The response rate will be defined as a decrease in AHI of at least 50% compared to baseline or an AHI under treatment of < 15 events/h. Post-hoc comparisons will be made based on these models, correcting for multiple testing by means of Bonferroni–Holm stepdown correction.

Non-inferiority of PSG compared to DISE will be judged based on the confidence intervals obtained from the mixed-effects models. When the lower limit of the confidence interval falls above the predefined non-inferiority margin, non-inferiority will be concluded. The non-inferiority margin for the percent change in AHI from baseline will be set at 10%. For the change in VAS for snoring, the ESS, the CIS20R and its fatigue subscale the margin is considered, respectively, to be 1, 2, 5 and 10 points. Additionally, differences between subjective titration and both RCMP titration protocols will be investigated for all of the secondary endpoints.

If more than 20% of all information regarding one endpoint is missing, a sensitivity analysis will be conducted for this endpoint using multiple imputation. Results of the original analysis will be interpreted in the light of these sensitivity analyses.

#### Randomization

This study design will lead to six different sequences of titration procedures (Table 1). The patients will be randomly allocated to one of the six possible sequences of titration procedures. To ensure that the groups are of approximately the same size, block randomization will be used. The allocation sequence will be automatically generated and will be stratified by OSA severity (moderate/severe), gender and body mass index (BMI < 26 kg/m^2^; 26 ≤ BMI < 30 kg/m^2^; 30 kg/m^2^ ≤ BMI).

**Table 1:** Overview of the six possible titration sequences in this study protocol

Sequence	Order of titration procedures		
1	Subjective titration	RCMP titration during PSG	RCMP titration during DISE
2	Subjective titration	RCMP titration during DISE	RCMP titration during PSG
3	RCMP titration during PSG	Subjective titration	RCMP titration during DISE
4	RCMP titration during PSG	RCMP titration during DISE	Subjective titration
5	RCMP titration during DISE	Subjective titration	RCMP titration during PSG
6	RCMP titration during DISE	RCMP titration during PSG	Subjective titration

*DISE* drug-induced sleep endoscopy, *PSG* type 1 polysomnography, *RCMP* remotely controlled mandibular positioner

## Discussion

Mandibular advancement devices are a valuable nonsurgical treatment option for patients diagnosed with OSA. The literature has shown that the amount of mandibular protrusion is a key factor in optimizing MAD efficacy and that the final titration of the protrusion should be carried out individually, trying to find the most effective protrusion, whilst at the same time respecting the patient’s physical limits [20, 39]. However, there is still no consensus on the optimal titration method.

The conventional approach relies on improvement of subjective symptoms, such as snoring and/or daytime sleepiness, for guidance of the titration toward the ideal mandibular protrusion in the individual OSA patient [24–27].

A more objective approach includes the use of an RCMP during a full-night PSG: in a process analogous to a CPAP titration night, the mandible is progressively advanced during sleep each time respiratory events occur throughout the night [21]. However, this is a time-consuming, labor-intensive and expensive procedure, because of the need for an overnight and attended hospital stay [12].

Civelek et al. [12] studied the effect of modulated CPAP pressure on upper airway collapsibility under direct visualization applying DISE. This DISE-assisted titration showed comparable results regarding the optimal CPAP pressure that alleviates OSA as compared to the conventional titration PSG. However, the titration procedure during DISE was found to be less labor-intensive and time-consuming as compared to a titration PSG [12].

The present randomized crossover trial will assess the mandibular protrusion values of MAD treatment under DISE-assisted titration using RCMP and compare these to the values obtained using a titration PSG, as well as with those obtained using the conventional subjective titration method. Furthermore, if different mandibular protrusion values are obtained according to the studied titration methods, it will be possible to compare the efficacy using level 1 sleep studies recorded at the end of each procedure.

Ideally, the same titration protocol for the DISE-assisted titration and the titration PSG is considered. However, due to the technical limitations of both procedures, some minor adaptations were made to the DISE-assisted titration protocol compared to the titration PSG as described earlier in the Methods section of this manuscript.

Firstly, as described by Remmers et al. [21], the RCMP titration PSG is scored afterward by an independent researcher based on predetermined rules: the predicted effective target protrusive position is the minimum protrusive position that was associated with ≤ 1 respiratory event per 5-min REM sleep. However, during DISE, propofol will be administered intravenously using a target-controlled infusion pump. This sedative drug is known to totally suppress REM sleep [40]. Therefore, during DISE, sedation to a level that is similar to stage 2 sleep is aimed at and examined by ensuring an absence of patients’ eyelash reflex after stimulation by means of a gentle brush. When target sedation is reached, RCMP will be remotely protruded in response to upper airway collapse until a stable upper airway together with an absence of oxygen desaturation and snoring is reached.

Secondly, during PSG the patient’s mandible will be protruded remotely in 0.5-mm steps in response to evidence of apneas and/or hypopneas. Similar protrusive steps can be applied during DISE-assisted titration.

However, this will require too much time since it is suggested to limit the maximal time for DISE with RCMP to 45 min to avoid ergonomic discomfort of the examiner, hypersalivation of the patient due to the trays, patient movements, arousals and submental tension possibly due to genioglossus muscle traction [30]. Therefore, in the DISE-assisted titration protocol, we opted to protrude the mandible in increments of 2 mm in response to upper airway collapse. If a stable upper airway is noted, the mandible will be retruded in 1-mm steps. This approach will be repeated until the effective target protrusive position can be determined, defined as the minimal mandibular threshold position of a stable upper airway in the absence of snoring, oxygen desaturation and apneas. When a stable upper airway is reached, the titration will proceed in 0.5-mm steps. By doing so, similar accuracy of the mandibular protrusion can be obtained during the RCMP titration DISE compared to the titration PSG.

To our knowledge, this study is the first to compare the mandibular protrusion values obtained during remotely controlled titration during DISE with the remotely controlled titration during PSG, as well as with the protrusion values obtained using conventional subjective titration. Furthermore, if different mandibular protrusion values are obtained with the different titration methods, it will be possible to compare the efficacy of each titration protocol using the polysomnographic evaluation recorded at the end of each protocol, to assess inferiority or non-inferiority of these titration procedures.

## Data Availability

Case report forms (CRFs) will be set up in the web-based software tool OpenClinica to capture clinical study data. The datasets used and/or analyzed during the current study are available from the corresponding author on reasonable request.

## Abbreviations

AHI: Apnea–hypopnea index
CIS20R: Checklist Individual Strength
CPAP: Continuous positive airway pressure
DISE: Drug-induced sleep endoscopy
EDS: Excessive daytime sleepiness
ENT: Ear, nose and throat
ESS: Epworth Sleepiness Scale
MAD: Mandibular advancement device
OSA: Obstructive sleep apnea
PSG: Polysomnography
RCMP: Remotely controlled mandibular positioner
REM: Rapid eye movement
VAS: Visual analog scale
