# Correction to: Remotely controlled mandibular positioning of oral appliance therapy during polysomnography and drug-induced sleep endoscopy compared with conventional subjective titration in patients with obstructive sleep apnea: protocol for a randomized crossover trial

**DOI:** 10.1186/s13063-020-04313-2

**Published:** 2020-04-17

**Authors:** Marijke Dieltjens, Marc J. Braem, Sara Op de Beeck, Anneclaire V. M. T. Vroegop, Elahe Kazemeini, Eli Van de Perck, Jolien Beyers, Chloé Kastoer, Kristien Wouters, Marc Willemen, Johan A. Verbraecken, Olivier M. Vanderveken

**Affiliations:** 1 Translational Neurosciences, Faculty of Medicine and Health Sciences, University of Antwerp, Wilrijk, Antwerp Belgium; 2 Department of Otorhinolaryngology, Head and Neck Surgery, Antwerp University Hospital, Wilrijkstraat 10, 2650 Edegem, Antwerp Belgium; 3 Department of Special Dentistry Care, Antwerp University Hospital, Edegem, Antwerp Belgium; 4 Multidisciplinary Sleep Disorders Centre, Antwerp University Hospital, Edegem, Antwerp Belgium; 5 Clinical Trial Center, Antwerp University Hospital, Edegem, Antwerp Belgium; 6 Department of Pulmonology, Antwerp, University Hospital, Edegem, Antwerp Belgium; 7 Laboratory of Experimental Medicine and Pediatrics (LEMP), Faculty of Medicine and Health Sciences, University of Antwerp, Wilrijk, Antwerp Belgium

**Correction to: Trials**

**http://dx.doi.org/10.1186/s13063-019-3698-4**

Following publication of the original article [1], the authors reported that Fig. 1 had not been corrected based on the reviewer’s comments. The correct Fig. 1 is presented below.

**Fig. 1:**
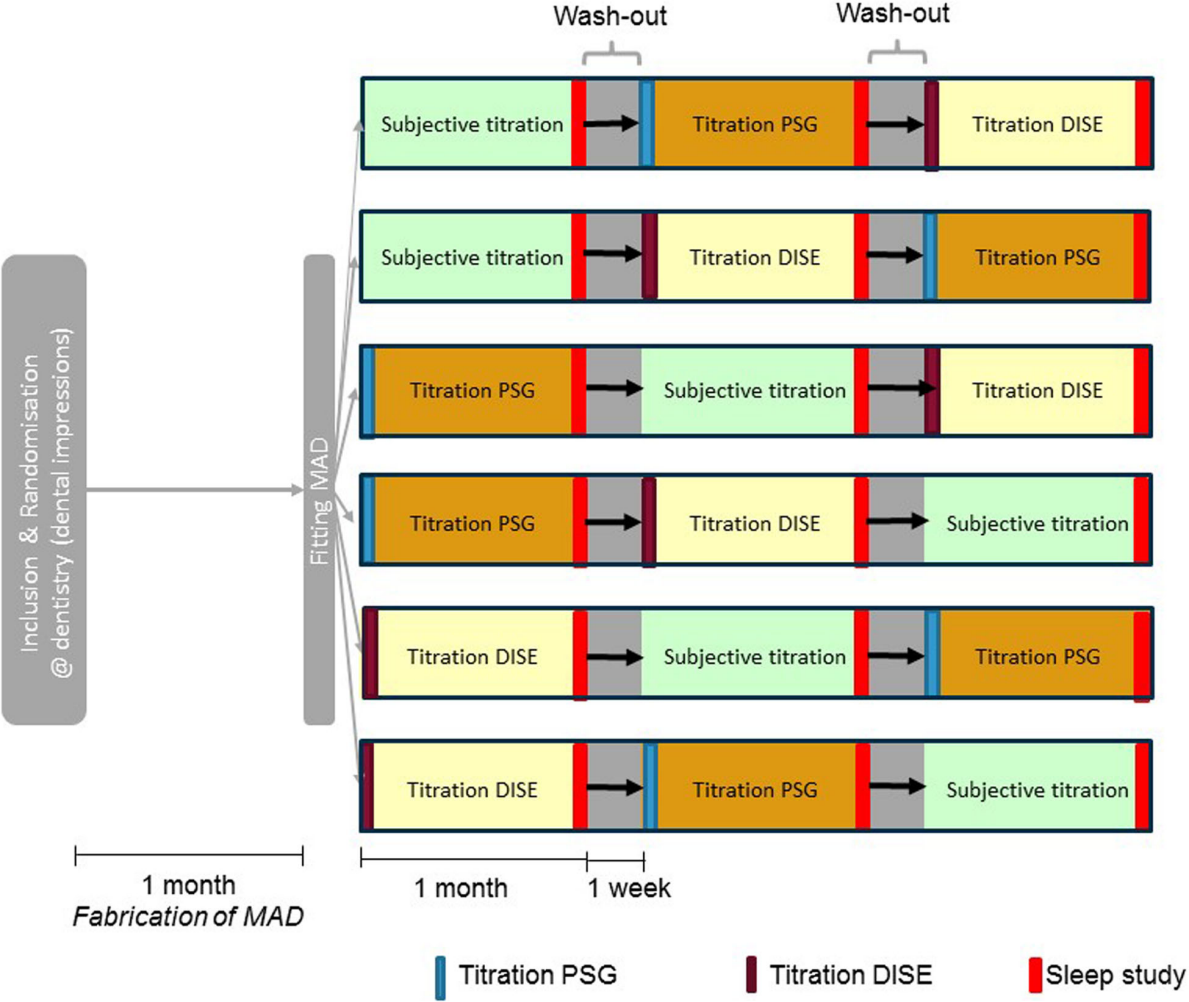
Schematic overview of the study design. Patients will undergo three titration procedures —subjective titration (green), titration PSG (orange) and, titration DISE (yellow) — in randomized order. This leads to six different possible sequences. A 1-week washout period is integrated between the different titration procedures. A follow-up sleep study is performed at the end of each titration method. DISE, drug-induced sleep endoscopy; MAD, mandibular advancement device; PSG, polysomnography
